# Self-Organizing Sub-μm Surface Structures Stimulated by Microplasma Generated Reactive Species and Short-Pulsed Laser Irradiation

**DOI:** 10.1021/acsomega.3c10033

**Published:** 2024-06-27

**Authors:** Sascha Chur, Lennart Kulik, Volker Schulz-von der Gathen, Marc Böke, Judith Golda

**Affiliations:** Plasma Interface Physics, Ruhr-University Bochum, 44801 Bochum,Germany

## Abstract

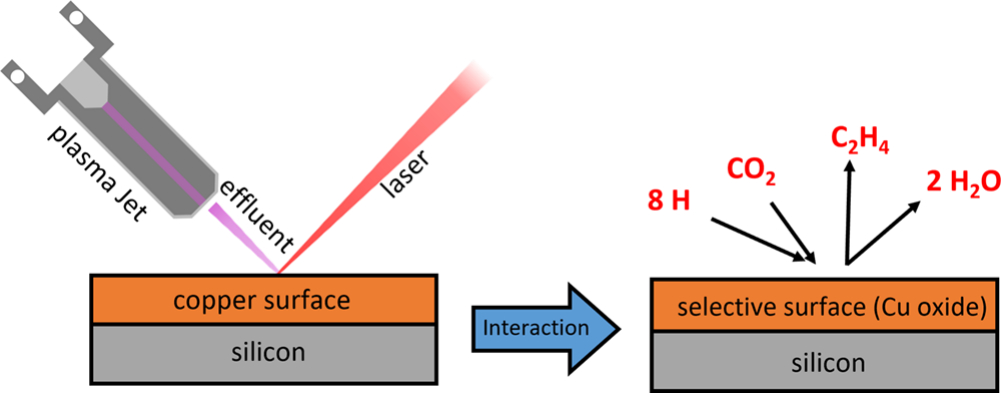

Catalysts are critical components for chemical reactions in industrial applications. They are able to optimize selectivity, efficiency, and reaction rates, thus enabling more environmentally friendly processes. This work presents a novel approach to catalyst functionalization for the CO_2_ reduction reaction by combining the reactive species of an atmospheric pressure plasma jet with the electric fields and energy input of a laser. This leads to both a nanoscale structuring as well as a controllable chemical composition of the surface, which are important parameters for optimizing catalyst performance. The treatment is conducted on thin copper layers deposited by high power pulsed magnetron sputtering on silicon wafers. Because atomic oxygen plays a key role in oxidizing copper, two photon absorption fluorescence is used to investigate the atomic oxygen density in the interaction zone of the COST plasma jet and a copper surface. The used atmospheric pressure plasma jet provides an atomic oxygen density at the surface in a distance of 8 mm to the jet nozzle of approximately  or a flux of . Pulsed laser-induced dewetting is used to form nanoparticles from the deposited copper layer to enhance catalytic performance. Varying the layer thickness allows control of the size of the particles. A gas flow directed on the sample during the combined treatment disturbs the particle formation. This can be prevented by increasing the laser energy to compensate for the cooling effect of the gas flow. Investigating the surface using X-ray photoemission spectroscopy reveals that the untreated copper layer surface consists mostly of metallic copper and Cu(I) oxide. Irradiating the sample only with the laser did not change the composition. The combination of plasma and laser treatment is able to produce Cu(II) species such as CuO, whose concentration increases with treatment time. The presented process allows the tuning of the ratio of C_2_O/CuO, which is an interesting parameter for further studies on copper catalyst performance.

## Introduction

Catalysts find use in a wide range of industrial and laboratory applications because of their ability to optimize reaction rates, selectivity, and energy efficiency of chemical reactions. They also play an important role in enabling more environmentally friendly processes in regards to energy consumption as well as waste material production.^1^ The efficiency of a catalyst is heavily influenced by its surface characteristics, like its morphology and chemical composition.^2^

An important and promising chemical process regarding green house gas emission reduction is the CO_2_ electroreduction reaction (CO_2_RR) to produce high value carbons.^1,3,4^ Enabling this process for industrial applications could achieve a sustainable carbon cycle.^5,6^ Because CO_2_ is a highly stable molecule, catalysts are needed to facilitate this process at lower temperatures, ideally provided by renewable energies.^2^ A promising catalyst for the CO_2_RR is copper (Cu), which is abundant and is comparatively cheap. Furthermore, it is the only metal catalyst found to be able to produce ethylene, ethanol and n-propanol (C_2+_ products) with substantial yields.^7−9^ However, it suffers from poor selectivity toward C_2+_ products and is unstable under electrocatalysis.^10,11^

Copper oxides were found to improve selectivity^12^ as well as performance.^11,13−16^ The two most commonly investigated copper oxides are Cu(I) oxide Cu_2_O and Cu(II) oxide CuO, which differ mainly in the reduction of the Cu atom, leading to different lattice structures and bonding with the O atoms. They were able to prolong the lifetime of the catalytic surfaces under CO_2_RR.^17,18^ Additionally Cu(II) species show a high selectivity toward C_2+_ products with over 50% but further research is needed to reach a comprehensive understanding of the processes.^19,20^ Especially CuO seems to have a comparatively high selectivity for ethylene production.^4,6^ Analyzing and influencing the catalyst’s chemical surface composition is therefore critical to understanding its catalytic performance.

Plasmas are able to produce highly reactive species like atomic oxygen to treat and activate surfaces by oxidizing them, for example. Reactive oxygen species enhance the copper oxidation and are able to produce Cu(II) species.^21^ In investigations of plasma activated catalytic surfaces were found to achieve higher performance in the CO_2_RR.^17,18,22^ As such, it is important to characterize the behavior of reactive oxygen species to understand their influence on the chemical surface composition of catalysts.

Another method for increasing the performance of catalytic surfaces is surface structuring. Particularly nanoparticles are able to improve selectivity and performance with regards to forming C_2+_ products.^23−25^ Surface structuring can be achieved by laser-induced surface structuring such as laser-induced periodic surface structures (LIPSS)^26^ or pulsed laser-induced dewetting (PLID).^27^ Pulsed laser-induced dewetting forms nanoparticles driven by the capillary forces of the melted metal film to reduce its large surface-to-volume ratio.^27,28^ Although controlling the input parameters is straightforward, it is able to produce complex patterns reliably, making it an interesting tool for nanoscale surface structuring.^29,30^

Laser irradiation is able to form self-organizing structures in the submicrometer range,^26,27^ which enhance catalysis.^2,31^ But it is not understood how laser irradiation will impact the oxidation of the surface or its chemical composition in general. Additionally, laser-plasma-surface interactions have not been studied previously to our knowledge. This is true, especially for the laser surface structure formation under plasma treatment. However, the combination of plasma treatment and laser irradiation may lead to an effective functionalization of the surface for catalytic performance due to the above-mentioned advantages to both treatments and could provide a more tunable process than commonly employed methods.

As such, we investigate a new approach to catalyst fabrication to control the chemical composition and surface morphology simultaneously by combining the reactive species produced by a microscaled atmospheric plasma jet and the irradiation and energy input of a pulsed laser on copper surfaces. To optimize the process, it is critical to investigate laser parameters such as pulse energy and power, as well as the reactive species flux to the treated sample.

## Experimental Setups and Methodologies

### COST Atmospheric Pressure Micro Plasma Jet

Because atomic oxygen is one of the main drivers for the oxidation of copper,^21^ understanding its behavior in interaction with the catalytic surface is critical. For the investigation of atomic oxygen, a thoroughly understood production source is needed. The COST microscale atmospheric pressure plasma jet^32^ is well characterized and is able to provide a high density of atomic oxygen and other reactive species. It is operated using a 13.56 MHz radio frequency with a plane electrode configuration. The discharge channel between the electrodes is 1 × 1 mm in diameter with two quartz glass planes on the sides to provide optical access to the discharge channel. Inside the jet various reactive species can be produced, which are then transported to a surface via the gas stream exiting at its nozzle, the effluent. In the following measurements, a polished copper surface was used.

### Two Photon Absorption Laser-Induced Fluorescence Spectroscopy

**Figure 1:**
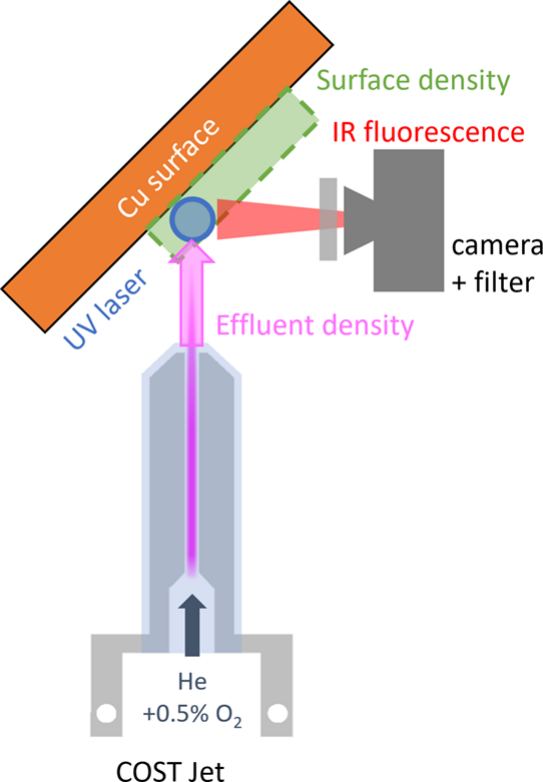
Jet to surface configuration for the two photon absorption laser-induced fluorescence (TALIF) setup. Pink and green markers visualize areas for effluent and surface measurements.

To investigate the atomic oxygen density distribution along the effluent between the COST jet and a copper surface, two photon absorption laser-induced fluorescence (TALIF)^33^ was used. The setup is displayed in Figure 1. The COST jet was standing upright, with the effluent pointing at the surface. The distance between copper surface and jet was approximately 8 mm, and the surface had a 45° angle to the jet effluent with the jet pointing upward. The laser was aligned parallel to the surface and perpendicular to the jet effluent. The laser system consisting of a Nd:YAG laser and a dye laser produced an excitation wavelength of 256 nm to excite atomic oxygen from the groundstate (O(2p^4 3^P)) to the O(3p ^3^P) state. For the detection of the 844 nm emission, an infrared sensitive ICCD camera with a resolution of 100 μm was used. It provided a better spatial resolution than the photomultiplier that was used before in Steuer et al.^34^ This allowed for decoupling of the effluent profile from the atomic oxygen density measurement.

### Sample Fabrication

The treated samples are silicon wafers with a copper layer deposited by high power impulse magnetron sputtering (HiPIMS). This provided better control over the surface characteristics. The better layer adhesion from the HiPIMS deposition also proved critical for the PLID particle formation, as layers deposited with direct current magnetron sputtering (DCMS) lead to an ablation of the layer under laser irradiation.

### Laser-Induced Surface Structure and Analysis

**Figure 2:**
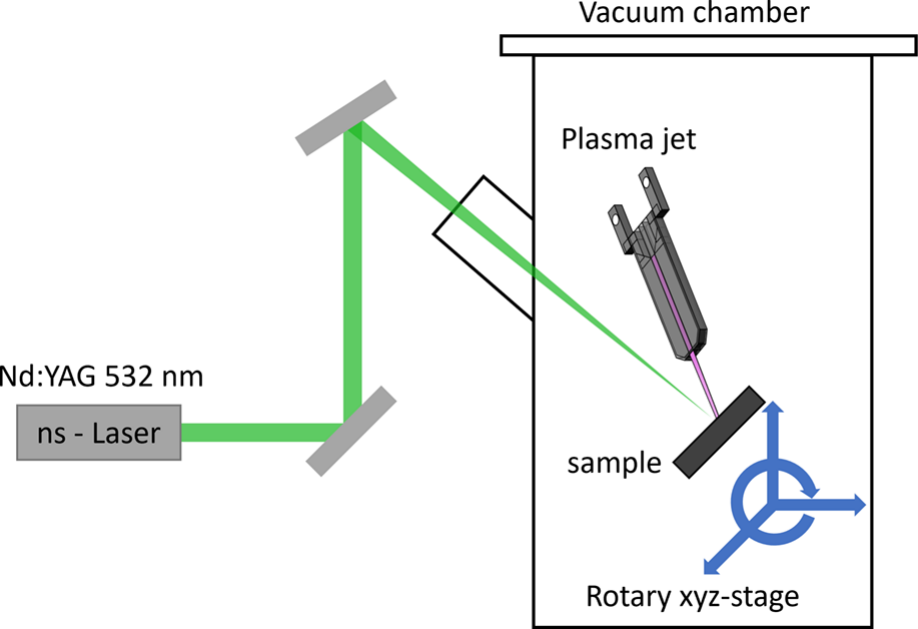
Setup for the sample treatment, combining plasma treatment and laser irradiation.

The surface treatment aims to combine irradiation with a laser and reactive species provided by a plasma jet. The experimental setup is shown in Figure 2. The laser is a frequency-doubled Nd:YAG laser with a wavelength of 532 nm running at 20Hertz. Beam quality enhancement by a pinhole provided a Gaussian laser energy distribution profile. Its energy and beam shape were measured by using a laser energy probe and a beam profiler (gentec-eo Beamage 3.0). The sample is mounted on a stage, which is able to move in all spatial directions and can be tilted at an angle with the laser beam. The sample was rotated so that the laser was aligned parallel to the sample surface normal. The generated spot on the samples where the laser was focused was typically around 400 μm in diameter, resulting in a laser fluence of  as a flat top approximation. The plasma jet effluent was lined up with the laser spot position on the sample to provide the gas stream directly to the area irradiated by the laser. The plasma jet was shifted 45° to the side from the position of the laser, thus hitting the surface at an angle of 45° degrees. The flow of helium and oxygen could be adjusted by using mass flow controllers. For the gas admixture, mostly 1 slm He with 0.5% O_2_ was chosen, as it provided the highest production of atomic oxygen, which plays a key role in the oxidation of copper.^35^ To reduce the effects of unknown reactive species in ambient air, the laser-plasma treatment was conducted inside a vacuum chamber with a controlled atmosphere at 980 mbar, using the same admixture as the gas flow.

### Surface Morphology Analysis

The laser-induced surface structures were investigated by using the scanning electron microscope (SEM) JEOL JSM-7200F. The SEM measurements used an accelerating voltage of 10 keV and a probe current of 1 nA at a working distance of 4 mm. Two surface morphologies could be observed after laser irradiation: laser-induced periodic surface structures (LIPSS)^26^ and pulsed laser-induced dewetting (PLID)^27^ (shown @@in 7). The dewetting of the copper on the silicon wafer formed nanoparticles, while LIPSS are wave shaped and are on the scale of the laser wavelength of 532 nm. Because the PLID structures are much smaller than LIPSS and nanoparticles were already shown to improve catalytic performance,^23^ they are more interesting from an application point of view.

### Chemical Composition Analysis

It was shown that copper oxides have favorable catalytic properties with regard to yield and selectivity for C_2+_ products. To investigate the effect of combining laser and plasma treatment on the copper oxidation state, the samples were investigated using X-ray photoelectron spectroscopy (PHI 5000 Versaprobe) regarding their Cu 2p and the CuLMM auger spectra. A quantitative analysis of the copper oxide concentrations using the Cu 2p spectra is difficult because the peaks of Cu metal and Cu(I) species as well as CuO and Cu(OH)_2_ have very similar binding energies and are not distinguishable from one another. Therefore, no deconvolution of the Cu 2p peaks was performed. Instead, we investigated the CuLMM auger spectra using the method described by Biesinger.^36^ The shape of the Auger spectra is distinct for every Cu species. When a sample contains multiple Cu species, the resulting Auger peak shape is considered to be a superposition of the reference spectra. The reference spectra were provided by Biesinger and were CuLMM Auger spectra taken from pure copper species samples. This approximation introduces an error for heterogeneous compositions of copper species because the background for the auger spectra is changing for composite states compared to the pure reference spectra.

To calculate the concentration, the CuLMM spectra were fitted in CasaXPS^37^ with the references, with their fitted area corresponding to their percentage. The fit restrains are shown in Table 1. The reference spectra were taken from Biesinger.^36^ Because the reference spectra had a fixed width of 16 eV, all Auger spectra were fitted for the range of 563 to 579 eV. Measurement and reference are both corrected with a linear background.

**Table 1:** Fit Restraints of the Cu Species Reference Spectra

Cu species	position constrains/eV
Cu metal	568.0, 567.8
Cu_2_O	569.9, 569.7
Cu(OH)_2_	570.3, 570.1
CuO	569.0, 568.8

## Results and Discussion

### Atomic Oxygen Density Distribution in the Effluent and on the Surface

To understand the influence of the plasma jet treatment on surface oxidation, the reactive species flux and its distribution in front of the surface have to be known. Atomic oxygen is a highly reactive oxygen species that is able to form copper oxide.^21^ TALIF measurements were performed to quantify the atomic oxygen density distribution produced by the COST jet in the interaction zone with a surface. The presence of a surface changes the gas dynamics. Therefore, it impacts the reactive species density distribution when compared to a free effluent, which was already studied extensively for the COST jet, e.g., by Steuer et al.^34^ In our application, we used an oxygen admixture of 0.5% for all measurements because it yielded the highest atomic oxygen production.

**Figure 3:**
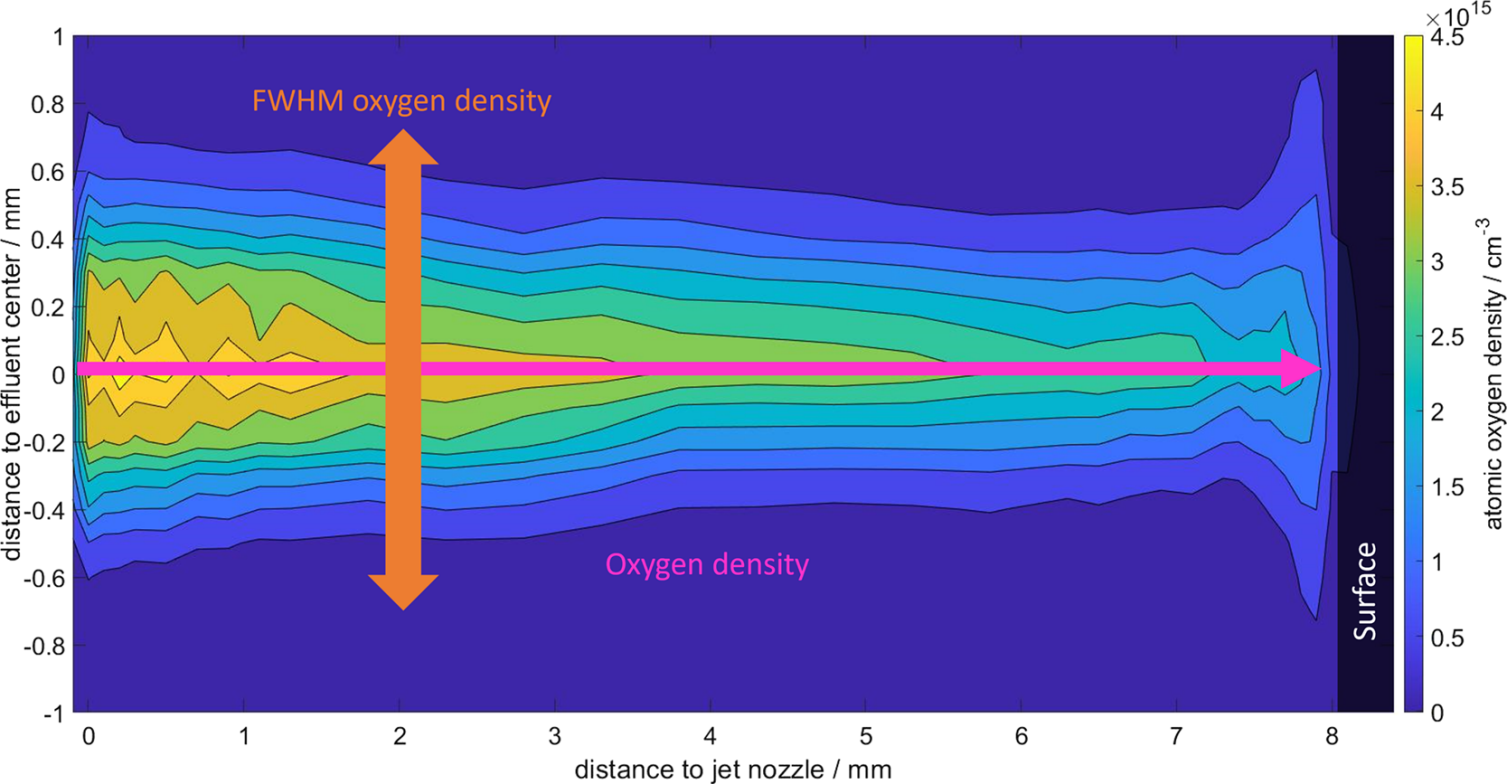
Two-dimensional atomic oxygen density distribution along the effluent (region shown in Figure 1) to the surface for a 2 slm He flow measured with TALIF. Gas admixture was helium with 0.5% oxygen. Markers visualize the density scan direction and the full width at half-maximum (fwhm) of the distribution profile.

Figure 3 shows the two-dimensional atomic oxygen distribution between the jet and the copper surface. The colored markers visualize the density measurement (pink) and effluent profile width (orange). The jet nozzle is on the left and the surface is on the right at approximately 8 mm distance to the nozzle. The density profile narrows with an increasing distance to the jet nozzle as the atomic oxygen density decays. In front of the surface, however, the distribution broadens when the gas stream hits the surface.

**Figure 4:**
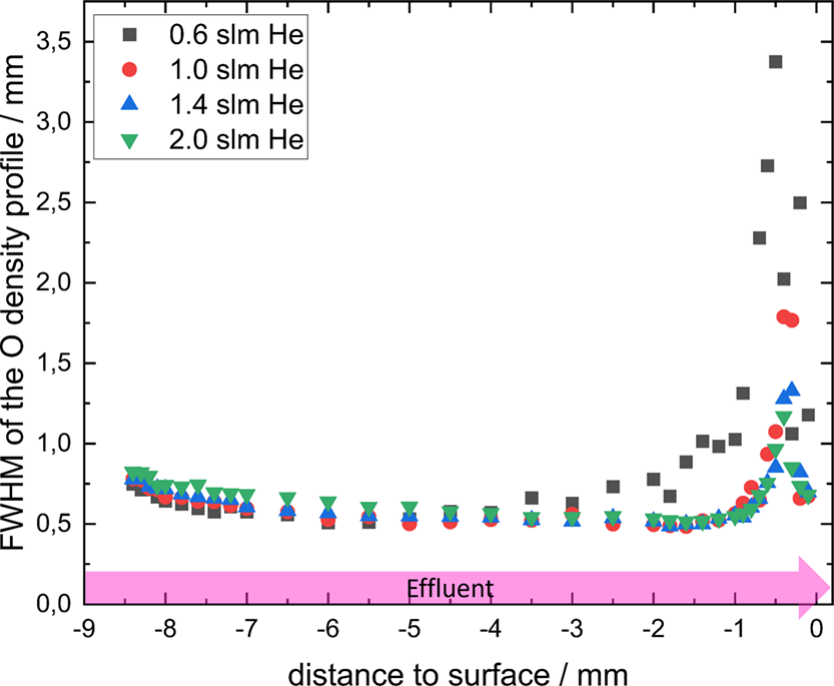
Full width half-maximum of the atomic oxygen density along the effluent (pink region shown in Figure 1) to the surface for different He gas flows. Gas admixture was helium with 0.5% oxygen.

The change in the width of the atomic oxygen density profile is depicted in Figure 4 for helium flows of 0.5, 1.0, 1.4, and 2.0 slm for a gas mixture of helium with 0.5% oxygen. The atomic oxygen density profile along the effluent to the surface was fitted with a Gaussian and then analyzed regarding its full-width half-maximum (fwhm). When leaving the jet nozzle, the fwhm for the different gas flows decreases slightly. Here ambient air is diffusing into the effluent, locally increasing the quenching at the edges. Near the surface, the fwhm increases because the gas flow is spread along the surface. This distributes the atomic oxygen over a larger area.

**Figure 5:**
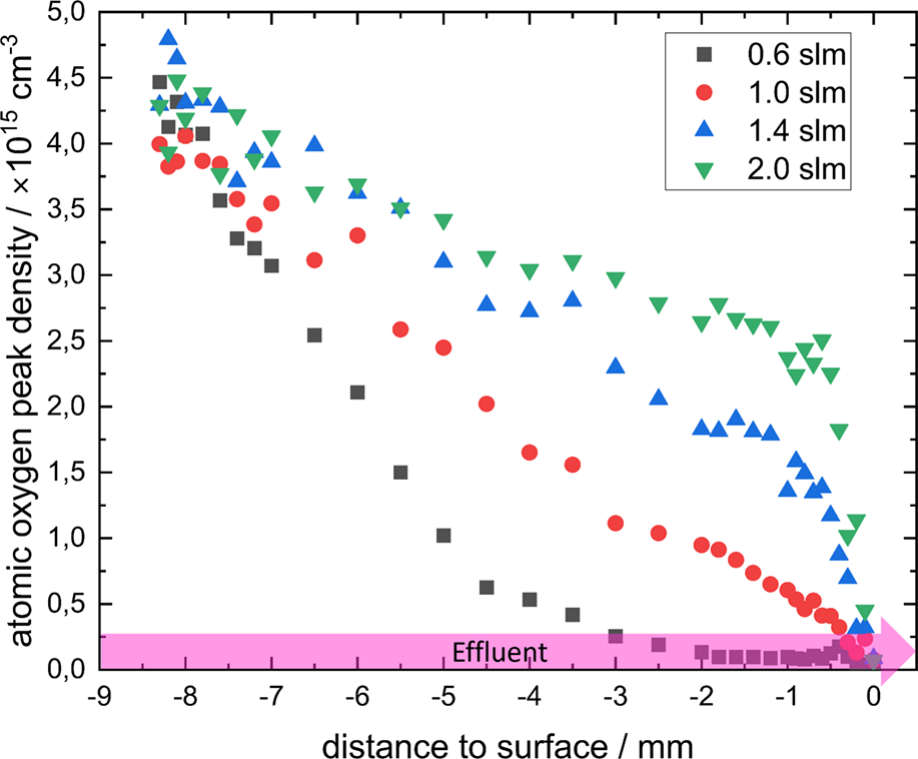
Atomic oxygen densities along the effluent (pink region shown in Figure 1) to the surface for different He flows measured with TALIF. Gas admixture was helium with 0.5% oxygen.

Figure 5 depicts the atomic oxygen densities for different gas flows along the effluent center to the surface. When a picture is taken with the ICCD camera the observed TALIF signal is a convolution of the laser width and the effluent expansion. The density describes the atomic oxygen density maximum of the observed detection volume, so the highest intensity pixel detected in the TALIF signal volume (located in the center of the effluent). This allows a decoupling of the effluent profile from the TALIF signal because the ICCD camera resolution is much smaller than the effluent width (100 μm to 1 mm).

The atomic oxygen density decreases with an increasing distance to the jet nozzle. Reactive species in the effluent normally show an exponential decay as observed in many studies.^38,39^ This is also true for atomic oxygen as shown in Steuer et al.^34^ as the atomic oxygen is quenched along the effluent. This behavior is visible for the lowest flow of 0.2 slm. For higher flows, the short observation length makes it difficult to identify the exact decay length. When comparing the atomic oxygen decay length with those measured by Steuer et al.,^34^ the decay length here is higher, especially for higher flows. This is due to the decreased effect of quenching in the center of the effluent. Ambient air is diffusing into the effluent from its edges, which has a higher quenching coefficient than helium. The center of the effluent remains free of air for a longer time and thus is quenched slower. Because the peak density is taken at the center of the effluent, this results in a higher atomic oxygen lifetime.

**Figure 6:**
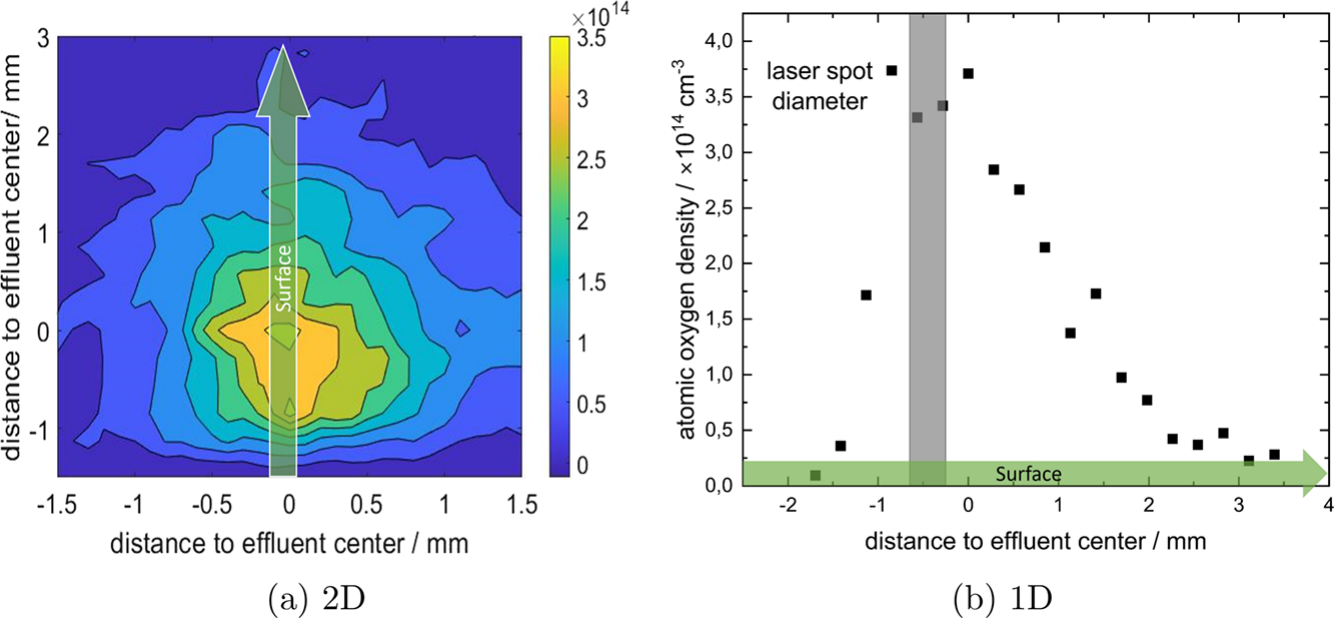
Atomic oxygen density distribution along the surface (green region shown in Figure 1) for a helium flow of 1 slm. The green arrow indicates the direction of the surface tilted away from the jet.

Figure 6a shows the atomic oxygen distribution directly along the surface when it is observed from the position of the jet. It illustrates the distribution of atomic oxygen over the surface. The profile shows a slightly asymmetrical exponential decay along the effluent flow direction axis due to the 45° tilt of the surface with regard to the plasma jet. This introduces a favored gas flow direction following the lower friction of the side angled away from the jet. This also coincided with the buoyancy direction of the helium. The asymmetry of the distribution along the surface was higher for lower gas flows, mainly because of the increased contribution of buoyancy on the helium flow. However, Schlieren imaging revealed that this favored gas flow direction was present for many different jet-surface configurations even when buoyancy was pointing in another direction (not shown here).

As depicted in Figure 6b, the spot size of the laser on the sample was 400 μm. In this range, the atomic oxygen density does not change considerately along the surface, so its distribution can be considered homogeneous over the laser spot. From the measurements, we can derive the atomic oxygen flux to the surface Γ by multiplying the density *n* with the gas velocity *v*: Γ_O_ = *n*_O_ * *v*. In the case of the COST Jet with a flow of 1 slm the gas velocity is . With an atomic oxygen density of approx  at the surface with a distance of 8 mm to the jet nozzle, the flux results in . This gives us an estimation of the amount of atomic oxygen delivered to the copper surface for oxidation, which is an important parameter for tuning the chemical surface composition.

### Laser-Induced Surface Structuring

Nanoscale surface structures can provide a significant enhancement in performance for catalytic surfaces.^23−25^ Plenty of research was conducted on the formation of laser-induced surface structures^27,28^ but the influence of a gas flow or reactive species provided by a plasma jet was not yet investigated to our knowledge. As both particle formation and reactive species integration into the treated surface are important to our approach, we had to investigate and possibly mitigate the effects of combining surface structuring and plasma treatment. To that end, we employed pulsed laser-induced dewetting to generate nanoparticles on the prepared samples.

The nanoparticle spacing and size of PLID can be described by the thin film hydrodynamic theory^27^ proposed by Trice et al. To validate this dependency for our samples, different copper layer thicknesses from 3 to 10 nm were irradiated with the laser until successful particle formation was observed. Although the optimal laser parameters depend on the film thickness, no notable changes in size and distribution occurred for treatment times of 5 to 20 s, which corresponds to 100 and 400 shots, respectively. Thus, for all thicknesses, a laser energy of approximately 0.56 mJ with an irradiation of 10 or 200 shots was used. The laser diameter was 400 μm.

**Figure 7:**
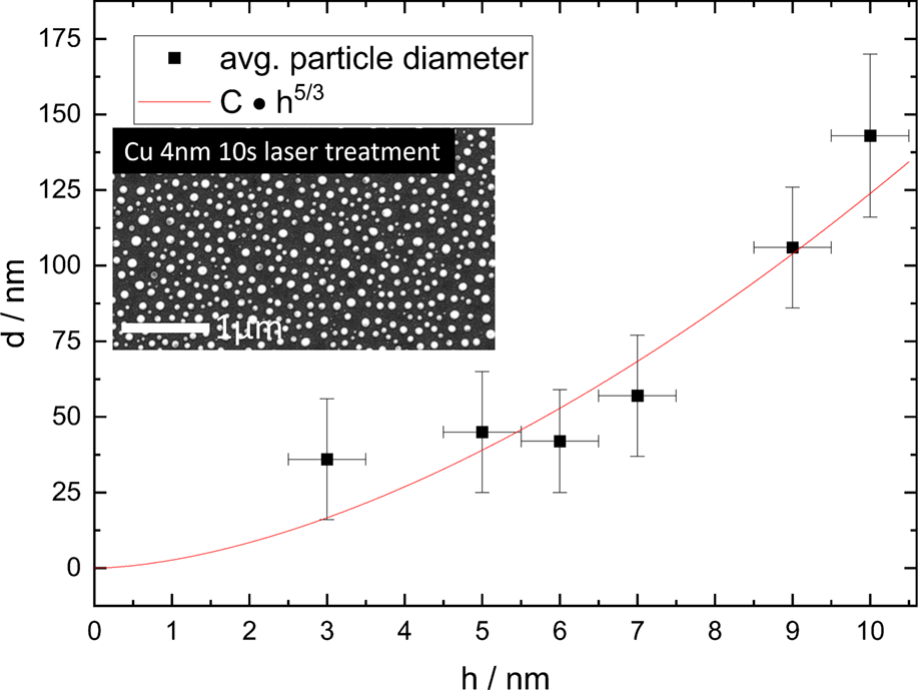
PLID nanoparticle diameter depending on the copper layer thickness, fitted using thin film hydrodynamic dewetting theory as described by Trice et al.^27^ Inserted picture shows PLID nanoparticles after 10 s of laser irradiation on a 4 nm copper layer on silicon.

The PLID surface structures were investigated by using secondary electron microscope imaging. The nanoparticles were then analyzed using ImageJ regarding their diameter, and their spatial distribution was fitted with a Gaussian to determine the mean diameter for a given copper layer thickness deposited on the silicon wafer. As seen in Figure 7, the diameter dependency follows the prediction given by the thin-film hydrodynamic dewetting theory, described by Trice et al.,^27^ very well. Under the assumption of a half-sphere and determining the mean distance to the closest neighbor and diameter of the particles an approximation of the surface roughness is possible: *R*_m_ = 8 ± 1 nm with a particle density of ρ = 3 ± 1 μm^–2^.

### Laser Surface Structuring Interaction with a Gas Flow

For our application, we combined surface structuring through laser irradiation with the effluent of an atmospheric pressure plasma jet. To the best of our knowledge, the interaction of PLID particle formation in the presence of a gas flow has not been studied extensively before. We observed that when a gas flow is introduced during the laser irradiation, the optimal parameters to successfully produce nanoparticles change. Because the morphology can influence the XPS analysis of the chemical surface composition, it is important to have comparable surface structuring for all treatment types. To this end, we varied the jet distance to the surface and the laser energy to investigate their influence on particle formation and find laser and gas flow parameters that achieve a reproducible surface structure for different treatment types.

**Figure 8:**
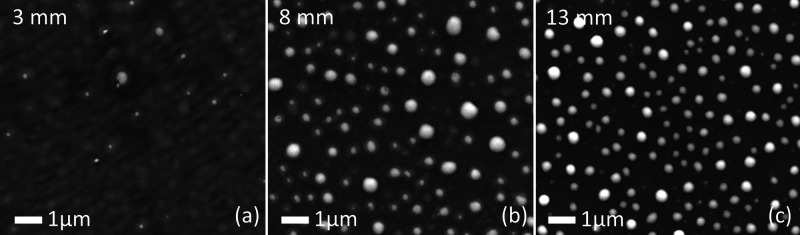
SEM images of the PLID nanoparticles for a laser energy of 0.56 mJ and 1 slm He gas flow to the sample with different jet distances: (a) 3 mm, (b) 8 mm, and (c) 13 mm.

Figure 8 shows SEM images of the surface structuring after laser irradiation with a laser energy of 0.56 mJ under a 1slm He gas flow for different jet distances. For the minimal distance of 3 mm (Figure 8a) no PLID structures could be observed. Instead mostly LIPSS or holes are formed. These changes in the behavior of the particle formation were attributed to the cooling of the copper layer during laser irradiation by the gas flow. For the PLID formation to work according to the thin-film hydrodynamic dewetting theory, the surface has to be instantly melted to prevent large temperature gradients in the layer.^27^ The helium gas flow onto the surface introduces additional cooling, which might lead to the layer not being completely melted during a laser pulse. This, in turn, prevents or disturbs PLID particle formation.

For 8 mm (Figure 8b), distinct particles can be observed, although they are clearly disturbed in their formation and appear “smeared” compared to the PLID nanoparticles without gas flow. Additionally to the above-mentioned cooling effect, the gas flow could disturb the droplet formation through friction. At a distance of 13 mm from the jet nozzle to the surface, nanoparticles are formed normally (Figure 8c). For these distances, the jet is far enough away so that the gas flow interaction with the sample is weak enough to not disturb the laser structuring process. This might be due to the decreasing gas velocity further away from the jet because of Stokes friction with the ambient atmosphere. Although particle formation is possible after a jet distance of 13 mm, the atomic oxygen measurements suggested, that with a flow of 1 slm the densities might be getting too low to have an effect on the chemical composition of the samples. This makes varying distance an ineffective control parameter.

**Figure 9:**
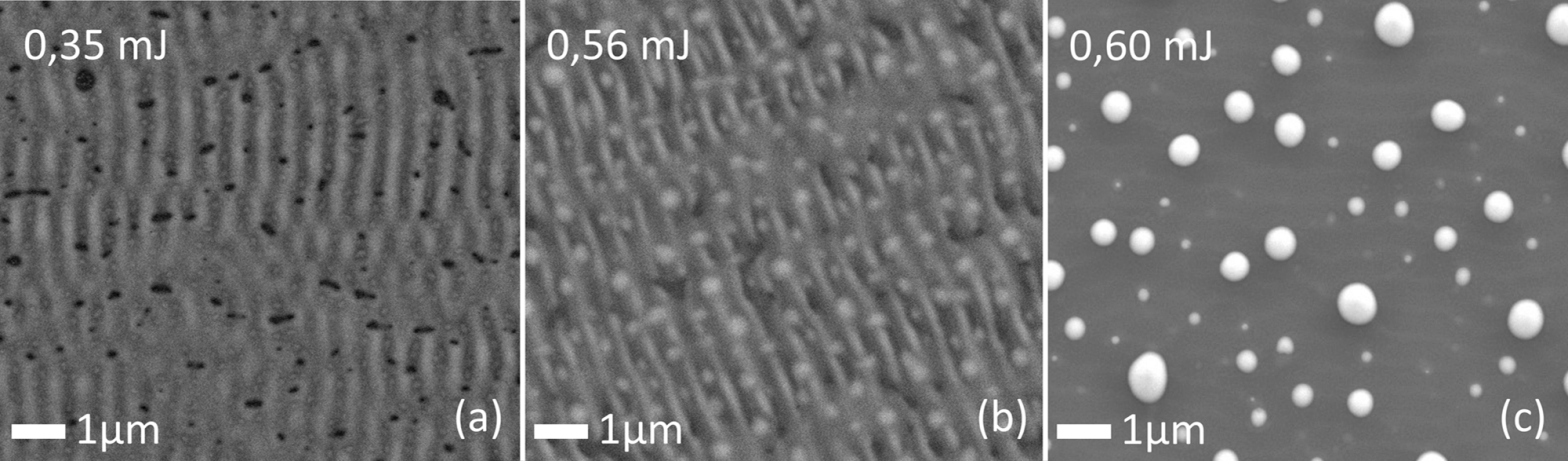
SEM images of the PLID nanoparticles for 10 nm Cu layer with 1 slm He gas flow and a jet distance of 8 mm to the sample for different laser energies: (a) 0.35 mJ, (b) 0.56 mJ, and (c) 0.60 mJ.

Increasing the laser energy could be a way of producing PLID nanoparticles, even with a gas flow present at smaller distances, by enabling the copper layer to melt completely during the laser pulse. Figure 9 shows the formation of different laser-induced structures with gas flow for different laser energies at a jet distance of 8 mm.

Low laser energies enable the formation of LIPSS as seen with Figure 9 for 0.35 and 0.56 mJ. Figure 9b shows an intermediate state between LIPSS and PLID formation, as the structure is largely reminiscent of the periodicity of LIPSS but clearly contains a few particle-like structures. The lower laser energies here might not be able to provide enough heat to the surface to melt it with the additional cooling of the gas flow. The low laser energy might not be able to provide enough heat to the surface to melt it completely with the additional cooling of the gas flow. LIPSS are a product of the interference of the laser light scattered by the surface roughness^26^ and do not require a completely molten copper for the laser structuring to work. Therefore, LIPSS are formed predominantly.

At 0.60 mJ, it was possible to fully form PLID nanoparticles. Therefore, a higher laser energy is able to achieve a similar surface morphology as the samples without a gas flow interaction. This is important for a comparison of the laser and laser-plasma treatment influence on the chemical surface composition as the properties are heavily influenced by the surface morphology.

### Effects of the Combined Laser and Plasma Treatment on the Chemical Surface Composition

Multiple studies have shown that the chemical composition of a surface, here specifically the oxides of copper, plays an important role for a catalyst by changing energy efficiency^11,13−16^ and selectivity.^12^ As such, an investigation of the chemical surface composition is critical in order to evaluate the surface regarding its catalytic performance. Moreover, it has to be examined what influence the laser and its surface structuring have on the oxidation of copper by the reactive species provided by the plasma jet. To analyze the samples, XPS measurements of the treated areas were performed.

**Figure 10:**
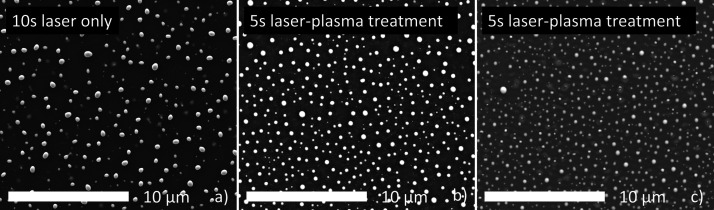
SEM images of the PLID nanoparticles for the 10 nm Cu layer on silicon for the different treatment types. (a) 0.56 mJ laser pulse energy for 10 s laser irradiation without gas flow, (b) 0.60 mJ laser pulse energy for 5 s with laser-plasma treatment, and (c) 0.60 mJ laser pulse energy for 10 s with laser-plasma treatment.

As XPS spectra are influenced by the surface morphology, it is critical to have a similar structure to be able to compare the effects of the different treatments. Figure 10 shows SEM images of the spots investigated with XPS. Image (a) shows the laser-treated surface, while (b) and (c) show the surface after combined treatment of plasma and laser for 5 and 10 s, respectively. The difference in particle size and distribution between the treatments is within the error shown in Figure 7 for a layer thickness of 10 nm and can thus be considered similar enough to not disturb the XPS analysis.

**Figure 11:**
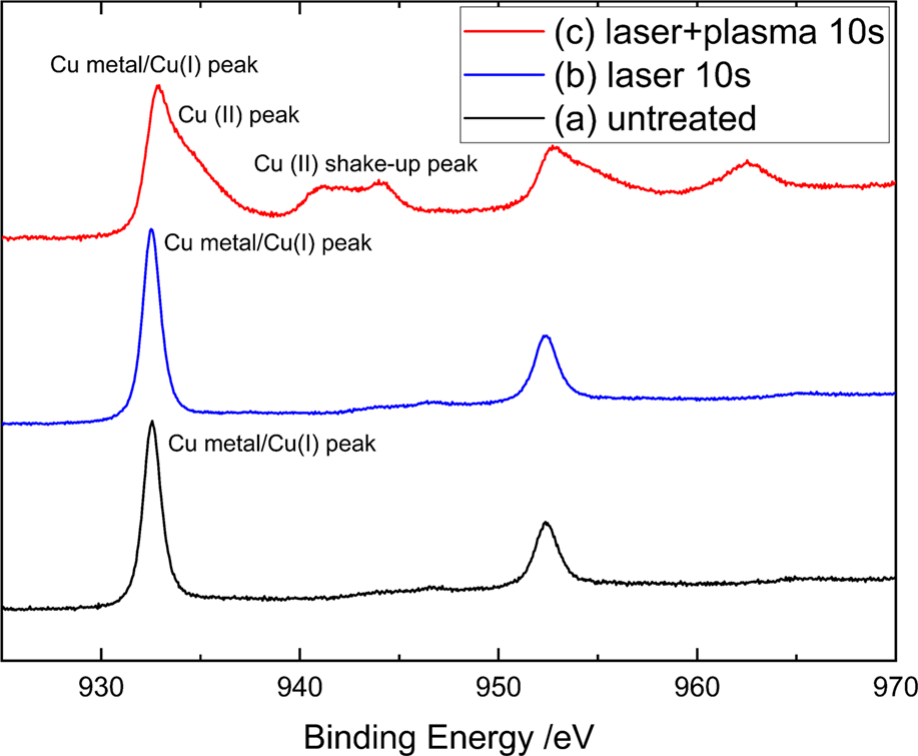
XPS Cu 2p spectra of the untreated Cu surface (a), the laser treated surface for 10 s (b), and the simultaneous treatment with plasma and laser for 10 s (c).

**Figure 12:**
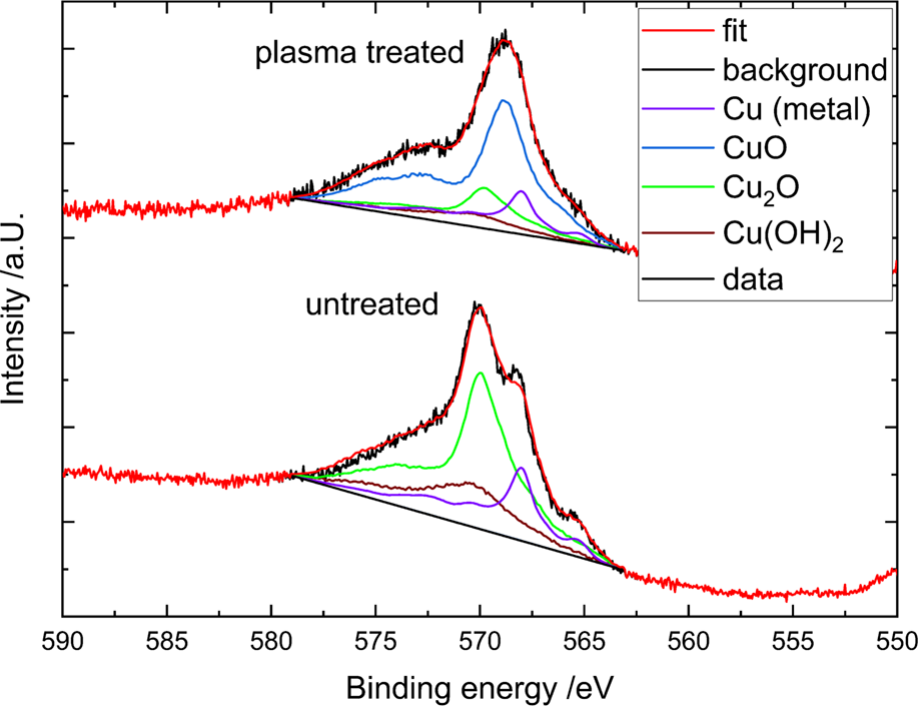
XPS CuLMM Auger spectra of the untreated Cu surface (bottom) and simultaneous treatment with plasma and a laser for 10 s (top).

Figure 11 shows the Cu 2p spectra of the untreated Cu surface as well as the laser-treated surface for 10 s and the surface treated with both plasma and laser for 10 s. The peak at the binding energy of approximately 932–933 eV indicates that the surface mostly consists of the Cu(I) species C_2_O and metallic copper. For the untreated and laser-treated surfaces, only the Cu (metal)/Cu(I) peaks of the Cu 2p_1/2_ and Cu 2p_3/2_ are visible. The XPS spectra for the only laser-treated surfaces show no notable changes in their Cu 2p (Figure 11), CuLMM (Figure 12), and O 1s (not shown here) spectra, when compared to the untreated surface. As such, it can be concluded that surface structuring with laser irradiation has no lasting influence on the chemical composition of the copper surface.

Applying the plasma treatment simultaneously with laser irradiation introduces a significant change in the surface oxidation state of copper. For the Cu 2p spectrum we can clearly see a shoulder peak emerging at a binding energy of 934 eV. In the Cu 2p spectra of the surface treated with both laser and plasma, broad peaks at 941–944 and 961–964 eV are visible. These are so-called shakeup peaks of the Cu 2p peaks and are the result of an ion excitation after the X-ray photoionization. This reduces the kinetic energy of the emitted electron. It is commonly observed in paramagnetic materials, which in this case are the Cu(II) species CuO and Cu(OH)_2_. These oxidation states were not present in the laser-treated and untreated samples, clearly indicating an effect of the plasma treatment. Because the binding energies of the metallic copper and Cu_2_O peaks are indistinguishable from the Cu 2p spectra, CuLMM auger spectra are used for quantitative analysis.

Figure 12 shows the auger spectra of the untreated surface and the surface treated with both laser and plasma for 10 s to show the difference in the peak shape for different copper species concentration ratios. The initial XPS spectra were fitted using a superposition of the reference spectra for the different copper species. For the untreated CuLMM spectrum, we see a double peak structure, which in our case results from the copper metal and Cu_2_O reference spectra. The binding energy of these species is wide enough apart (ca. 2 eV) to make them distinguishable. The Cu(OH)_2_ auger reference spectra also contribute to the fit, although the Cu 2p spectra do not indicate the presence of Cu(II) species (Cu(OH)_2_), due to their lack of a shoulder peak at 934 eV and the missing shakeup peak. This is not an effect of the plasma as there are no OH species present but could be because of impurities in our chamber during production or due to the oxidation of treated samples when they get into contact with ambient air during transfer to the XPS. Such a high concentration is still questionable as it would be visible in the Cu 2p spectra.

This disagreement was observed for samples that contained a heterogeneous copper species composition. They were found to contain a high amount of copper hydroxide, according to the fit of the reference spectra, without the Cu 2p spectra indicating any Cu(II) species. The high copper hydroxide concentration might be an error of the fit procedure as the binding energies get shifted due to the changing background of heterogeneous compositions that can not be described by the superposition of the reference spectra. This could skew the fit toward copper hydroxide to compensate for discrepancies between fit and measurement for higher binding energies. It would also have been favorable to investigate the reference spectra with the same XPS device or better even before every measurement to prevent errors due to different charge corrections or other effects caused by the use of different XPS devices.

Another effect could be the silicon support or the nanoparticle shape. It was shown that the interface of different materials is able to shift the observed binding energies.^40^ X-rays are also able to influence the chemical composition by degradation of copper species,^41^ although because of the comparatively short time frames, this might not play a significant role in our case.

**Figure 13:**
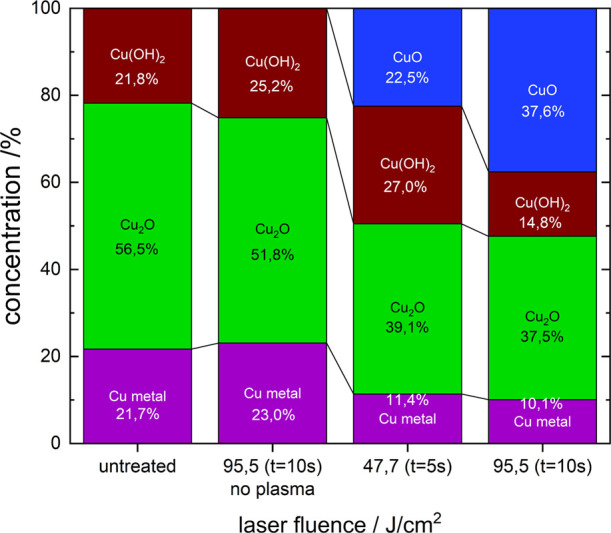
Concentration of the oxides CuO and Cu_2_O, the hydroxide Cu(OH)_2_ and metallic Cu depending on the laser fluence and treatment time.

With the CuLMM spectra, a quantitative analysis of the effect of the laser surface structuring and the reactive species flux on the surface composition is possible. Figure 13 shows the copper species composition for different treatment types and times compared with the untreated sample. For the untreated sample, the surface concentration of the Cu(I) oxide is roughly 57% with only a small deviation of 5% measured across multiple samples. The same is true for the metal copper and copper hydroxide concentrations, with the former at approximately 20% and the latter at 23%. This composition changes only slightly when the sample is treated only with the laser. Here the metallic copper and copper hydroxide percentages grow slightly to 23% or 25% for the latter. The Cu_2_O concentration is reduced to 52%. These changes remain within the error for the composition though.

When comparing the concentration distribution of the different Cu species for the untreated or only laser-irradiated samples with a combined laser-plasma treatment of 5 s, a clear reduction for the metal copper from 22 to 11% is visible as well as Cu_2_O from 53 to 39%. This reduction corresponds roughly to the increase in CuO of 23%. As such, it seems reasonable that CuO is formed mainly from further oxidizing metallic copper and Cu_2_O. The Cu(OH)_2_ also increased to 27%. This could be an oxidation effect as it is also a Cu(II) species but might also be the increased error due to the higher heterogeneity of the sample with the addition of CuO.

For a treatment time of 10 s, the concentration of metal copper stays nearly the same as for 5 s at 10%, while CuO increases from 23 to 38%. The Cu(OH)_2_ concentration decreases significantly from 27 to 15%, while Cu_2_O and Cu metal remain approximately the same from 39 to 38% for the former and 11 to 10% for the latter. From the 5 to 10 s treatment Cu(OH)_2_ is converted to CuO instead of metallic copper and Cu_2_O. It would be expected that metal copper and Cu_2_O get converted completely before also Cu(OH)_2_ decreases, as they are more easily oxidized. Cu(OH)_2_ on the other hand should be more stable regarding oxidation as a Cu(II) species. The lower Cu(OH)_2_ concentration might also be due to the higher percentage of CuO, decreasing the heterogeneity of the sample composition. This shifts the peak toward lower binding energies, lowering the discrepancy for the high binding energy flank of the fit compared to the measurement.

The formation of the different Cu oxides can be attributed to different reactive species provided by the plasma jet, as shown in eqs 1 to 5.
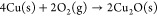
1
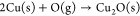
2
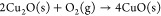
3
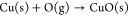
4
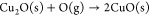
5

As Gusakov et al.^21^ have shown, atomic oxygen alone is not able to produce Cu(II) species and mainly forms Cu_2_O in contact with a copper surface. Only when molecular oxygen is present, Cu(II) and, in particular, CuO is formed. However, the slow reaction rate of ground-state molecular oxygen makes it an unlikely candidate for this reaction. Here excited molecular oxygen species such as O_2_(a ^1^Δ_*g*_) and O_2_(b ^1^Σ_*g*_^+^) may be responsible. They have been shown to possess reaction rates several magnitudes higher than that of ground-state molecular oxygen.^42,43^ The laser provides additional energy to the surface by heating. Gusakov et al.^21^ have shown that the absorption of oxygen into a copper surface is enhanced at higher temperatures. However, it should be noted that in the aforementioned study, lower temperatures were more favorable for the formation of CuO, while for high temperatures mainly Cu_2_O is formed. We clearly reach the melting temperature of copper at 1357 K, which is way above the temperatures found by Gusakov et al. However, the heating of the laser takes place over a 10 ns pulse, which is too short for the oxidation reactions to take place. After the laser pulse, the spot is rapidly cooled down so that the copper becomes solid. As such, after a pulse, the temperatures in the laser spot might reach values more favorable for the CuO formation but still high enough to enhance oxygen absorption into the surface. The Cu(OH)_2_ is most likely formed on the sample when it comes into contact with air during the sample transport to the various diagnostics.

Although longer treatment times are able to increase the CuO percentage, the range of possible treatment times is restricted by the laser. Shorter irradiation does not lead to the desired surface structuring, while longer irradiation ablates material from the sample, interfering with chemical modification of the surface. Further studies would need to be conducted for these edge cases.

## Conclusions

This work presented a novel approach to catalyst fictionalization, combining the reactive species of an atmospheric pressure plasma jet with the irradiation and energy input of a laser. The treatment was conducted on thin copper layers deposited by high-power pulsed magnetron sputtering on silicon wafers. Because atomic oxygen plays a key role in oxidizing copper, especially Cu(II) oxide species, two-photon absorption fluorescence was used to investigate the atomic oxygen density in the interaction zone of the COST plasma jet and a copper surface. For a distance of 5 mm and a flow of 1 slm He + 0.5% O_2_ the COST jet provides a density of approximately  or a flux of .

Laser irradiation is able to form nanoscale surface structures on treated surfaces, particularly nanoparticles, which can lead to enhanced catalytic performance. The modified surface morphology was investigated by using a secondary electron microscope. The underlying process for the applied surface structuring is pulsed laser-induced dewetting, which was shown to be able to control the nanoparticle size by varying the layer thickness following the thin film hydrodynamic instability theory presented by Trice et al.

However, combining the laser surface structuring with the gas flow of the jet disturbed the particle formation. This was, on the one hand, attributed to the interaction of the stream with the melted metal during laser irradiation. Although the effect could be compensated by increasing the distance to the jet, this procedure is inefficient because it reduces the atomic oxygen density reaching the surface. On the other hand, the gas stream provided additional cooling of the surface, preventing a complete melting of the copper layer. It was shown that increasing the laser power could overcome the cooling effect, resulting again in nanoparticle formation. The density is homogeneous over the laser diameter. Achieving a similar surface structure for different treatment types is important for a comparison of their chemical composition.

X-ray photoemission spectroscopy was used to investigate the chemical composition of the surface with special regard to the concentration of copper oxides. Radiating the sample only with the laser did not change the ratio compared to that of the untreated surface. Without plasma, mostly Cu(I) species were observed in the Cu 2p spectra, with only a small amount of copper hydroxide present in the CuLMM auger spectra. The combination of plasma and laser treatment was able to produce Cu(II) species CuO, whose concentration was shown to increase with the treatment time. Thus, the ratio of Cu_2_O/CuO can be controlled.

This study shows that the laser is able to structure the surface while the plasma is still able to influence the chemical surface composition in a controllable way. Being able to tune the CuO to Cu_2_O ratio is a unique trait of this method and is potentially interesting for further studies on complex copper catalysts.

Modifying surface morphology and chemical composition simultaneously and precisely makes this process usable for different applications, especially in research where defined conditions are needed for the investigation of catalytic performance. The mixed Cu_2_O/CuO state may also lead to an increased lifetime under the right conditions. The PLID particles are not able to agglomerate, which is a common problem of traditionally fabricated nanoparticles that reduces their efficiency. Because of these reasons, the presented surface design could be a step toward a more stable catalyst material.
